# Large-Area, Highly Sensitive SERS Substrates with Silver Nanowire Thin Films Coated by Microliter-Scale Solution Process

**DOI:** 10.1186/s11671-017-2351-y

**Published:** 2017-11-03

**Authors:** Sooyeon Jang, Jiwon Lee, Sangin Nam, Hyunhyub Ko, Suk Tai Chang

**Affiliations:** 1 School of Chemical Engineering and Materials Science, Chung-Ang University, Seoul, 06974 Republic of Korea; 2 School of Energy and Chemical Engineering, Ulsan National Institute Science & Technology (UNIST), Ulsan, 44919 Republic of Korea

**Keywords:** Silver nanowires, Thin film coating, Microliter-scale solution process, Surface-enhanced Raman spectroscopy

## Abstract

A microliter-scale solution process was used to fabricate large-area, uniform films of silver nanowires (AgNWs). These thin films with cross-AgNWs were deposited onto Au substrates by dragging the meniscus of a microliter drop of a coating solution trapped between two plates. The hot spot density was tuned by controlling simple experimental parameters, which changed the optical properties of the resulting films. The cross-AgNW films on the Au surface served as excellent substrates for surface-enhanced Raman spectroscopy, with substantial electromagnetic field enhancement and good reproducibility.

## Background

Surface plasmon resonance (SPR) is the collective oscillation of conduction band electrons on a metal surface excited by incident light at a metal-dielectric interface [1–3]. For nanostructures of noble metals such as gold and silver, the SPR absorption band is present in the visible region, and its exact wavelength is very sensitive to particle size, shape, spacing, and the surrounding dielectric medium [4, 5]. In particular, when two nanoparticles are close to each other with a nanoscale gap, the electromagnetic field is confined in this gap [6, 7], also known as a “hot spot.” Many efforts have been studied to reliably produce surface-enhanced Raman spectroscopy (SERS) hot spots, through the use of metal nanoparticle aggregates [8, 9], patterned arrays of nanostructures [10, 11], and metal films over nanospheres [12, 13]. This allows for highly sensitive SERS sensing systems, but their application is limited by the ability to fabricate structures with regular gap dimensions, which is a current challenge in nanofabrication.

Silver nanowires (AgNWs) have been studied as an ideal SERS candidate due to their large surface area, high phase purity, and good crystallinity [14]. For single nanowire studies, surface etching of AgNWs [15] and decorated metallic nanoparticles on AgNWs [16] have been shown to increase the amount of SERS active “hot spots.” To further increase these enhancements, AgNWs have been paired (crossed and parallel) [17, 18] and bundled [19] to create gaps between neighboring nanowires, increasing the electromagnetic fields present. AgNWs have been assembled into large surface area parallel arrays [20, 21], which showed strong SERS enhancements in the gaps between parallel AgNWs. While parallel arrays of AgNW films have been extensively studied, large-scale crossed AgNW assemblies have received less attention.

Homogeneous SERS substrate can provide uniform distributions of hot spots for single molecule detection. Many routes have been proposed to fabricate SERS-active nanostructures, such as Langmuir-Blodgett assembly [20], layer-by-layer assembly [22–25], convective assembly [26, 27], and electron-beam lithography [28–30]. However, some of these techniques are expensive, complex, and time-consuming, whereas others are not suitable for large-scale production of uniform SERS substrates.

Herein, we present a simple and scalable approach to fabricate high-density cross-patterned AgNW films on Au surfaces by utilizing a meniscus-dragging deposition (MDD) method. AgNWs were aligned in the coating direction while the deposition plate was moved back and forth, dragging the meniscus of a microliter of AgNW solution injected into the gap between the moving deposition plate (on top) and the Au substrate (on the bottom). To produce a large number of SERS hot spots, we fabricated cross-junctions between the nanowires by rotating the pre-coated substrate by 90° and repeating the process, resulting in uniform cross-AgNW films. In this study, we demonstrated that the cross-AgNW films show higher Raman intensity than drop-AgNW films of the same surface density. In particular, the cross-AgNW films on Au films shows 1.8 times stronger SERS enhancement than drop-AgNW films.

## Experimental

### Fabrication of Cross-AgNW Films

**Fig. 1:**
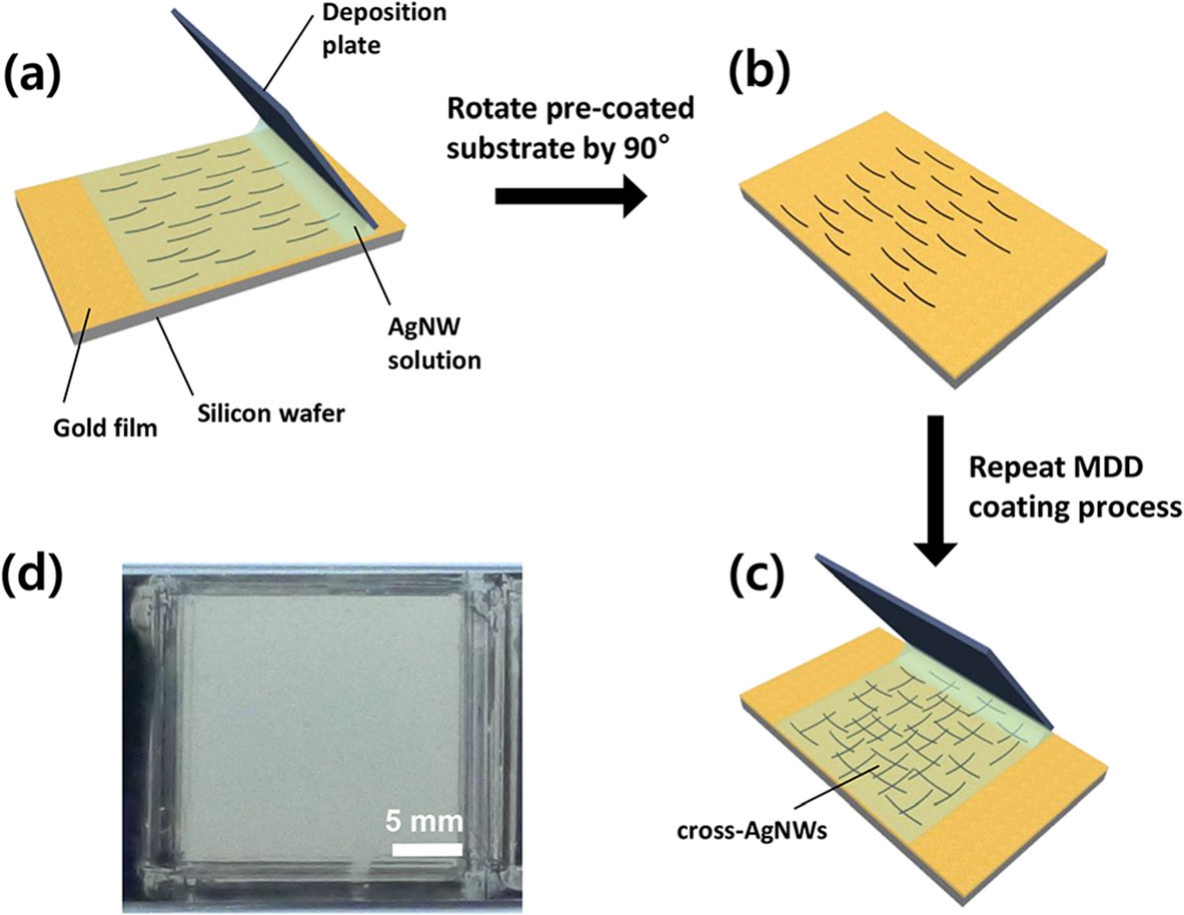
**a**–**c** Schematic illustration of MDD coating process for fabricating the cross-AgNW films on a gold surface. **d** Photograph of the cross-AgNW films with deposition number of 18

Silicon wafers (P/Boron, 1–30 Ω cm, 525+/−25 μm, Wafer Biz) were treated with piranha solution (H_2_O_2_:H_2_SO_4_ = 1:1) to produce a hydrophilic surface. To fabricate an Au substrate, an Au film (50 nm) was deposited onto a pre-cleaned silicon substrate by thermal evaporation deposition. A suspension of AgNWs (0.5 wt%) in isopropanol (IPA) was purchased from Sigma Aldrich. The average diameter and length of the AgNWs were approximately 60 nm and 10 μm, respectively. To produce high-density cross-AgNW films, the purchased AgNW/IPA suspension was concentrated to 1.5 wt% AgNWs by evaporating IPA in the 0.5 wt% AgNWs solution on a hot-plate at 100 °C for 30 min. Preparation of high-density cross-AgNWs was achieved using an MDD method [31–33] as follows: glass slides (25 × 75 mm^2^ with plain ends, Fisher Scientific) were treated with piranha solution for 30 min, rinsed in DI water, and dried before coating. Then, 2 μL of the 1.5 wt% AgNW solution was injected between the glass slide and the prepared Au film substrate, in contact with each other at an angle of θ = 30°. The deposition plate was moved back and forth using a motorized stage (AL1-1515-3S, Micro Motion Technology) at a rate of 20 mm/s to cover a 2 × 2 cm^2^ section of the Au film substrate. As the deposition plate was moved, the IPA was dried and the AgNWs became aligned with the shear stress applied by the moving plate (Fig. 1a). To fabricate a cross-array of AgNWs (Fig. 1c), the substrate with the as-deposited film was rotated by 90° (Fig. 1b), and this process was repeated. AgNW films were also prepared on Au substrates by drop-casting using the same concentrated AgNW/IPA suspension as a control sample.

### Characterization of Cross-AgNW Films

The fabricated Au/cross-AgNW films were characterized using digital photography (Lumix DMC-LX5, Panasonic), field emission scanning electron microscopy (FE-SEM, Carl Zeiss SIGMA), and UV-vis-NIR spectrophotometry (V-670, Jasco). To perform SERS using the prepared substrates, Au/cross-AgNW films were heated on a hot-plate at 110 °C for 10 min to remove the polyvinylpyrrolidone (PVP) layer on the AgNW surface. The SERS substrates were then dipped in 100 mM benzenethiol in ethanol (Sigma Aldrich) for 15 min, rinsed with ethanol, and then dried under N_2_. Raman spectra of benzenethiol were collected using a confocal Raman microscope (Alpha 300, WITec) with a 785 nm excitation laser. The integration time was 0.5 s, and the laser power was ~ 15 mW. Raman spectral images (40 × 40 μm^2^) were obtained under 15 mW laser power and 0.2 s integration times.

## Results and Discussion

To fabricate cross-patterned AgNW assemblies on Au film substrate, we used a MDD method as shown in Fig. 1. The concentrated AgNW/IPA suspension was injected between the deposition plate and the Au film contacted at an angle of θ = 30°, and a meniscus was formed between the end of deposition plate and the Au surfaces due to capillary action (Fig. 1a). When the deposition plate moved back and forth, the shear stress applied to the AgNWs in the meniscus causes them to assemble parallel to each other and align along the direction of the shearing force. After this process, the AgNW film substrate was rotated 90° (Fig. 1b), and another layer of AgNW was assembled on top of it (Fig. 1c). This process was repeated to form a high density of cross-AgNW assemblies with 8–18 layers. Using multiple deposition steps, we fabricated high density cross-AgNWs on Au film substrates, where 8, 10, 14, and 18 deposited layer samples are denoted as C-8, C-10, C-14, and C-18, respectively. The photograph in Fig. 1d shows the high-density AgNW assemblies on Au film with 18 deposition number, covering a relatively large area (2 × 2 cm^2^).

**Fig. 2:**
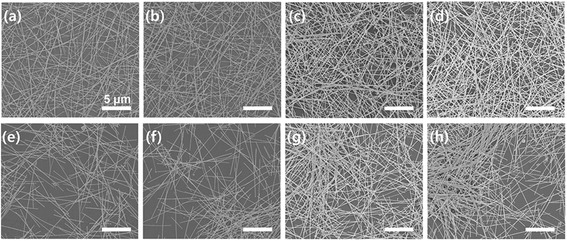
FE-SEM images of AgNW films with different deposition numbers and prepared by different coating methods. **a**–**d** The cross-AgNW films with different surface concentrations of AgNWs. **e**–**h** The drop-casted AgNW films with corresponding AgNW concentrations. Surface density of AgNWs on Au substrate: **a**, **e** 4.7 μg/cm^2^, **b**, **f** 5.9 μg/cm^2^, **c**, **g** 8.3 μg/cm^2^, and **d**, **h** 10.6 μg/cm^2^

To compare the performance of our cross-AgNW films to random-AgNW films, we fabricated four different surface densities of irregular AgNW films by drop-casting, such that the surface density of AgNWs was controlled by the concentration of the AgNW suspension. The different surface densities of the drop-casted AgNW films were defined by D-8, D-10, D-14, and D-18, corresponding to C-8, C-10, C-14, and C-18 above, respectively. The calculated surface densities of the AgNWs are 4.7 μg/cm^2^ (C-8, D-8), 5.9 μg/cm^2^ (C-10, D-10), 8.3 μg/cm^2^ (C-14, D-14), and 10.6 μg/cm^2^ (C-18, D-18). Figure 2 shows FE-SEM images of the cross-AgNW films (Fig. 2a–d) and random-AgNW films (Fig. 2e–h). The cross-AgNWs films show highly uniform cross-networks over the entire surface area, evident even at low magnification levels. In addition, the films became denser at increased deposition numbers and show increased numbers of AgNW junctions. On the other hand, images of random-AgNW films show both locally aligned and randomly deposited morphologies.

**Fig. 3:**
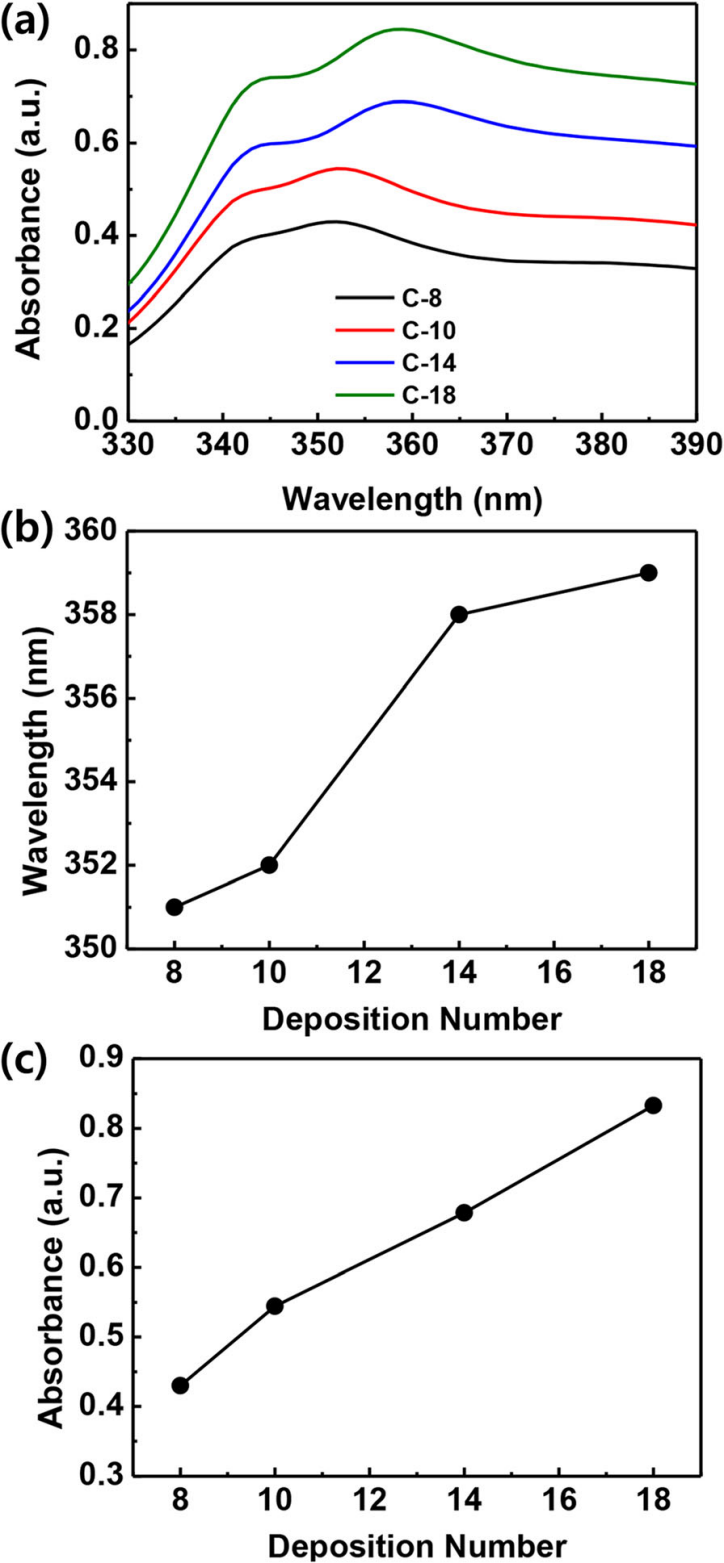
**a** UV-vis absorption spectra of cross-AgNW films with different deposition numbers. **b** Shift in resonance wavelength as a function of deposition number. **c** Absorption intensities at the maximum SPR (351–359 nm) peaks depending on deposition number

AgNWs enable a very intense light absorption band in the visible region. Figure 3 shows the UV-vis absorption spectra of cross-AgNW films on Au films with various deposition numbers. As can be seen in Fig. 3a, two maximum absorption peaks were detected, those being a weak peak at 343 nm and a broad peak at 351–359 nm. By increasing the number of AgNW conjunctions, the broad SPR peak is red-shifted from 351 to 359 nm (Fig. 3b). In addition, the absorption intensity of the SPR band gradually increases at increased surface densities (Fig. 3c). These results indicate that high-density AgNW films can lead to high light absorption by multiple plasmon couplings between neighboring AgNWs (crossed and parallel gaps) and between the Au film and the AgNW films.

**Fig. 4:**
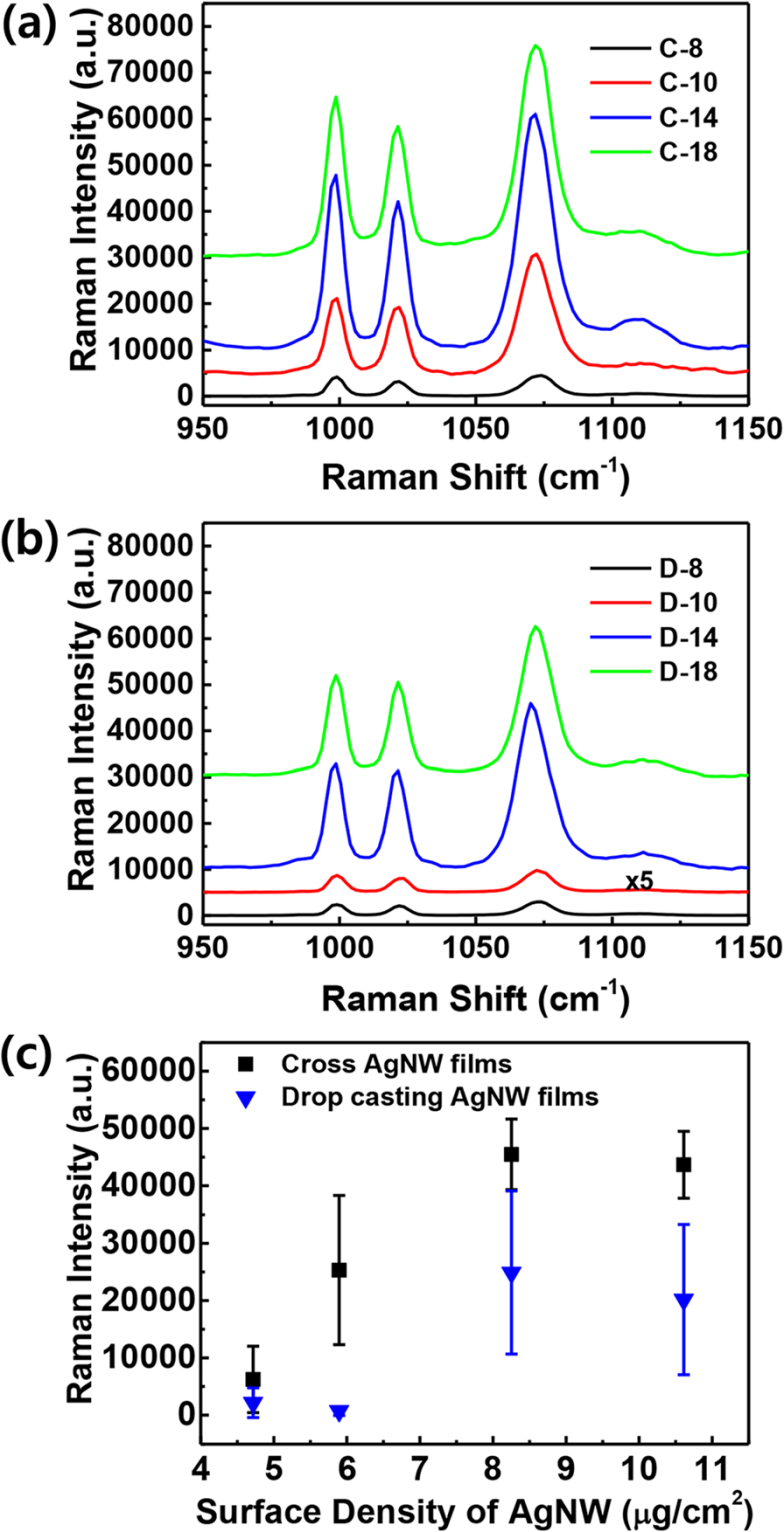
Raman spectra of benzenethiol on **a** the cross-AgNW films and **b** the drop-casted AgNW films coated on the Au surface. **c** Relative Raman intensities of the benzenethiol peak at 1071 cm^−1^ as a function of AgNW surface density

Raman intensities were compared between cross-AgNW films and drop-casted AgNW films incubated in 100 mM benzenethiol (Fig. 4). The Raman spectra of benzenethiol exhibit an in-plane ring-breathing mode (998 cm^−1^), an in-plane C-H bending mode (1021 cm^−1^), and an in-plane ring-breathing mode coupled with a C-S stretching mode (1071 cm^−1^) [34]. The SERS intensity of the cross-AgNW films increases with the AgNW surface density up to C-14, as shown in Fig. 4a. However, the SERS intensity of the C-18 sample was lower than that of the C-14 sample despite the high surface density of AgNWs, as strong inter-nanowire plasmon couplings shielded the propagating surface plasmon (PSP) of the Au film surface [35, 36]. The D-14 drop-casted AgNW films exhibits higher Raman intensity than the D-18 drop-casted AgNW films for this same reason (Fig. 4b). From these results, we can conclude that a suitable surface density of AgNWs is required for the amplification of SERS intensity. The C-14 and D-14 samples have the same surface density of AgNWs (8.3 μg/cm^2^) on the Au film, suitable for producing strong SERS intensity in samples prepared by both coating methods. However, the cross-AgNW films exhibited 1.8–36-fold higher SERS intensity than the drop-casted AgNW films because of geometric differences between the uniformly coated AgNWs (cross-AgNW films) and the partially aggregated AgNWs (drop-casted AgNW films), as shown in Fig. 4c. Consequently, the SERS intensities were affected by the array forms of AgNWs on Au films, and strong SERS intensity was created on the cross-AgNW films.

**Fig. 5:**
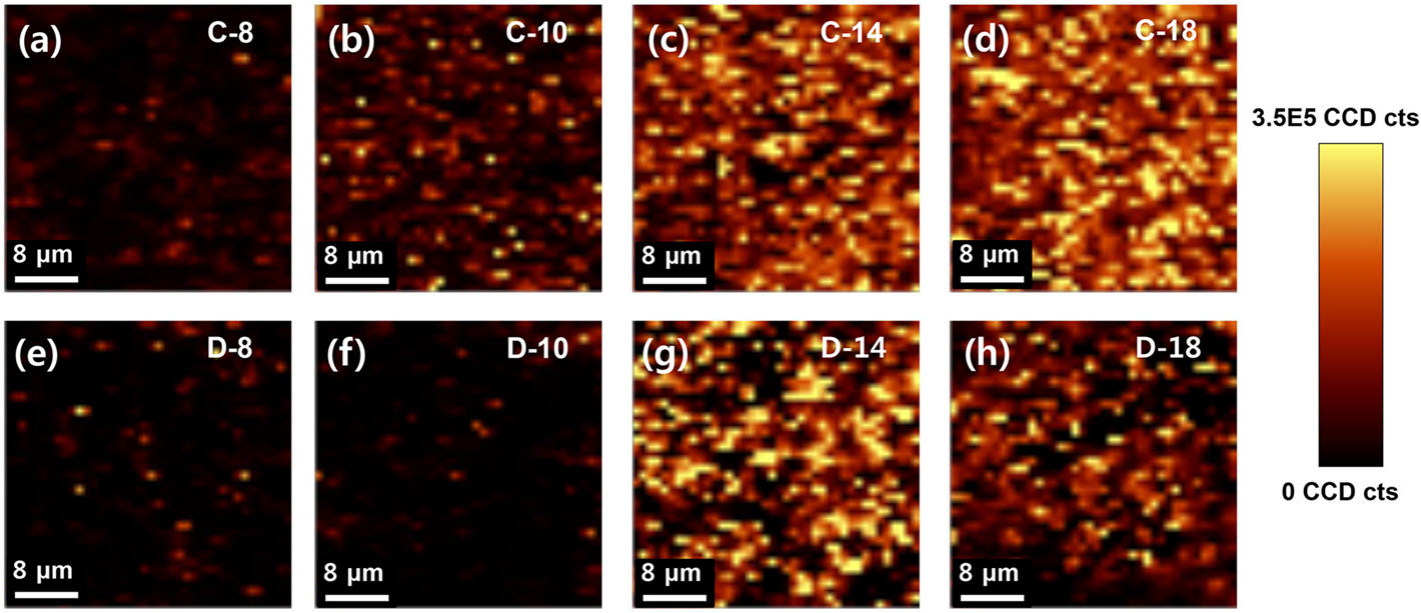
Raman spectral images of **a**–**d** cross-AgNW films on the Au surface with different surface concentrations of AgNWs and **e**–**h** drop-casted AgNW films on the Au surface with corresponding surface concentrations. Surface concentration of AgNWs on Au substrate: **a**, **e** 4.7 μg/cm^2^, **b**, **f** 5.9 μg/cm^2^, **c**, **g** 8.3 μg/cm^2^, and **d**, **h** 10.6 μg/cm^2^

Raman mapping was performed to study the homogeneity and spatial distribution of the integral area of Raman intensity at the 1071 cm^−1^ band of benzenethiol. The Raman spectral images in Fig. 5 show SERS hot spots on the Au-AgNW films. The reliability and reproducibility of Raman intensity quantification can be determined by counting these hot spots. As the number of layers increases, the Raman intensity increases and the spatial distribution of the Raman intensity becomes more homogeneous. In addition, the cross-AgNW films show regular and strong hot spots over the entire surface, but the drop-casted AgNW films were covered with randomly distributed hot spots. Therefore, the cross-AgNW films showed more uniform and stronger SERS intensity than the drop-casted AgNW films. In particular, C-14 (Fig. 5c) and C-18 (Fig. 5d) showed more hot spots than D-14 (Fig. 5g), demonstrating that the cross-AgNW films generated a larger number of hot spots than the drop-casted AgNW films for strong SERS enhancement.

## Conclusions

In summary, we have presented solution-based fabrication of extremely enhanced and reproducible large-area SERS substrates with uniform cross-arrays of AgNWs on Au; these arrays were produced using microliter volumes of AgNW suspension. The AgNWs were aligned by the shear stress applied to the meniscus of a droplet of AgNW suspension injected between the deposition plate and the coating plate. The regularly assembled AgNW films demonstrated better structural homogeneity and SERS intensity 1.8–36-fold higher than random, drop-casted AgNW films. The increased SERS intensity was attributed to an increase in SERS multiple plasmon couplings among AgNWs (crossed and parallel gaps) and between the Au film and the AgNWs. We have demonstrated that the SERS enhancement brought about by the cross-AgNW films was optimized at C-14 (Au/cross-AgNW films). Therefore, the cross-AgNW-based SERS substrate is sufficient to fabricate a highly sensitive SERS system. This approach has great potential for use in a wide range of applications in optoelectronics, nanoelectronics, and sensors.
